# Inadvertent Apical Extrusion of Sodium Hypochlorite with Evaluation by Dental Volumetric Tomography

**DOI:** 10.1155/2015/247547

**Published:** 2015-03-25

**Authors:** Elif Delve Başer Can, Meriç Karapınar Kazandağ, Rabia Figen Kaptan

**Affiliations:** Department of Endodontics, Faculty of Dentistry, Yeditepe University, Bagdat Caddesi 238, Göztepe, 34728 Istanbul, Turkey

## Abstract

This case report describes the tissue injury caused by inadvertently extruded NaOCl through the apical constriction. A 56-year-old female patient with complaints of pain, swelling, and ecchymosis on the left side of her face was referred to our clinic. The symptoms had emerged following root canal treatment of the maxillary left first premolar, and a soft tissue complication due to apical extrusion of NaOCl was diagnosed. Antibiotics and analgesics were prescribed. DVT images revealed that the buccal root apex had perforated the maxillary bone. The patient was followed up every other day and became asymptomatic on the 10th day. Endodontic therapy was completed with routine procedures. Determining working length precisely and following irrigation protocols meticulously are indispensable to prevent this type of complication. 3D visualization of the affected area may reveal the cause of the incident.

## 1. Introduction

Removal of bacteria and bacterial toxins from the root canal system during shaping and cleaning is the key factor for the success of endodontic therapy [1, 2]. Mechanical instrumentation is known to be insufficient to clean ramifications and anatomical irregularities [3, 4], and one-third of root canals remain untouched despite the advanced technology utilized in root canal instruments [5]. Therefore, mechanical preparation should be supported by a chemically active antibacterial irrigation solution [6]. The mechanical effects of irrigation are removal of microorganisms/biofilm, dentin debris, pulp tissue, and instrumentation products, whereas the chemical effects are dissolution of soft tissue remnants and smear layer and elimination of bacteria and their byproducts. A chemically active agent is required to achieve the chemical effects [7, 8].

Sodium hypochlorite (NaOCl) is the most popular irrigation solution to date, as it fulfills the majority of the required criteria [1, 9]. In contemporary practice, various concentrations of NaOCl (0.5%–6%) are used for root canal irrigation. The antibacterial and tissue dissolving effects of NaOCl are known to occur faster at higher concentrations [10]. It has been reported that 5.25% NaOCl was strong enough to kill the bacteria commonly present in the canal; however this concentration of NaOCl was highly toxic and irritating. Furthermore, 0.5% NaOCl dissolved the necrotic tissue but had no effect on* Staphylococcus aureus* [11]. The toxicity of NaOCl is due to its high alkalinity (pH 10.8–12.9) and hypertonicity [12, 13]. It causes oxidation of protein and lipid membrane and causes necrosis, hemolysis, and dermal ulcerations [14]. Discoloration of fabrics, ophthalmic injuries due to eye contact, apical extrusion, tissue emphysema, and allergic reactions are possible complications during irrigation of root canals with NaOCl [15].

Apical extrusion of an irrigation solution can occur when the pressure of the solution is excessive or if an irrigating needle is stuck in the root canal during shaping. Apical extrusion is likely to occur in teeth with larger apical diameters as well as a lack of apical constriction due to root resorption [15]. Common symptoms reportedly associated with NaOCl accidents are pain, swelling, ecchymosis, hemorrhage, and allergic reactions [16–21].

The purpose of this report is to describe the destructive effect of NaOCl solution on soft tissues following its extrusion during endodontic therapy and to analyze the cause of injury using dental volumetric tomography (DVT).

## 2. Case Presentation

A 56-year-old female patient with an unremarkable medical history visited our clinic with complaints of swelling, ecchymosis, and pain on the left side of her face. The dental history revealed that an endodontic retreatment therapy had been initiated on her maxillary left first premolar tooth 10 days previously, which was followed by a second session on the previous day. During the first session, the patient had felt light swelling, which resolved spontaneously a few hours later. At the second appointment, the treatment had to be stopped because of severe pain and hemorrhage from the root canal during irrigation of the root canal. No attempt had been made to restore the tooth other than placing a cotton pellet into the endodontic cavity. Another appointment had been scheduled by the general dentist to complete the treatment. The patient noticed swelling in her cheek several hours following the procedure, but she did not contact her doctor. However, her facial swelling worsened significantly over the next 24 hours, and the general dentist referred her to our clinic.

**Figure 1:**
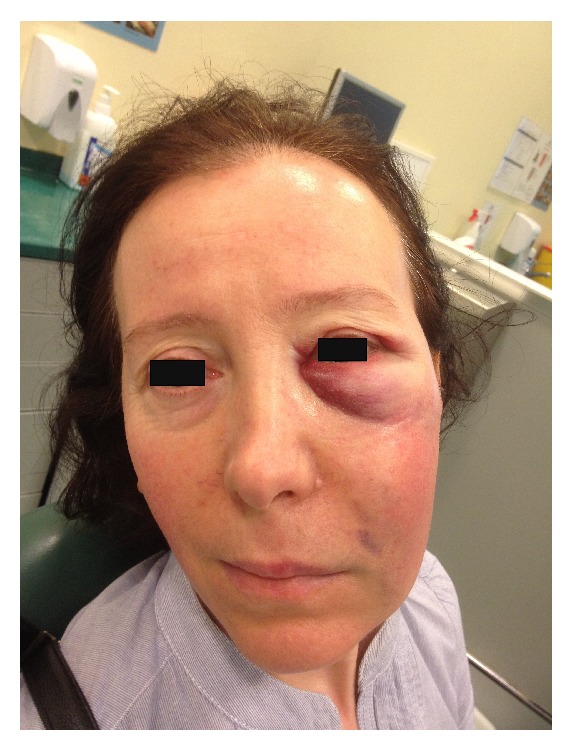
Infraorbital ecchymosis and slight bruising near the nasolabial fold.

**Figure 2:**
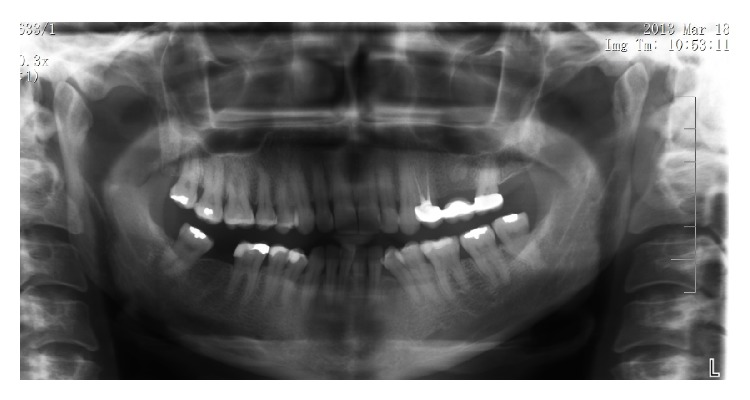
Panoramic radiography showed evidence of a previous root canal therapy with periapical radiolucency.

Approximately 48 hours after the incident, extraoral examination revealed significant soft tissue swelling extending from the left infraorbital region to the mandibular border. Infraorbital ecchymosis and slight bruising near the nasolabial fold were observed (Figure 1). Intraorally, there were no signs of mucosal ulceration or necrosis. The tooth being treated was tender to vertical and horizontal percussion. Panoramic radiography showed evidence of a previous root canal therapy with periapical radiolucency (Figure 2).

**Figure 3:**
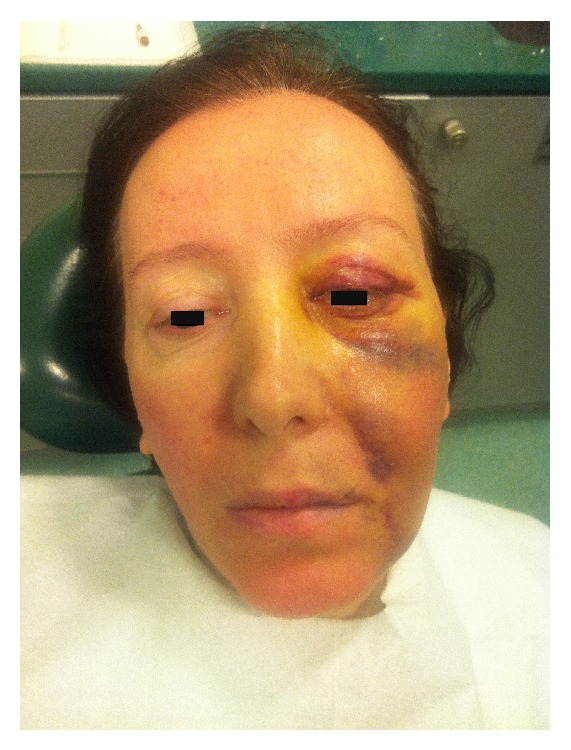
An increase in the ecchymosis was noticed on the first recall.

**Figure 4:**
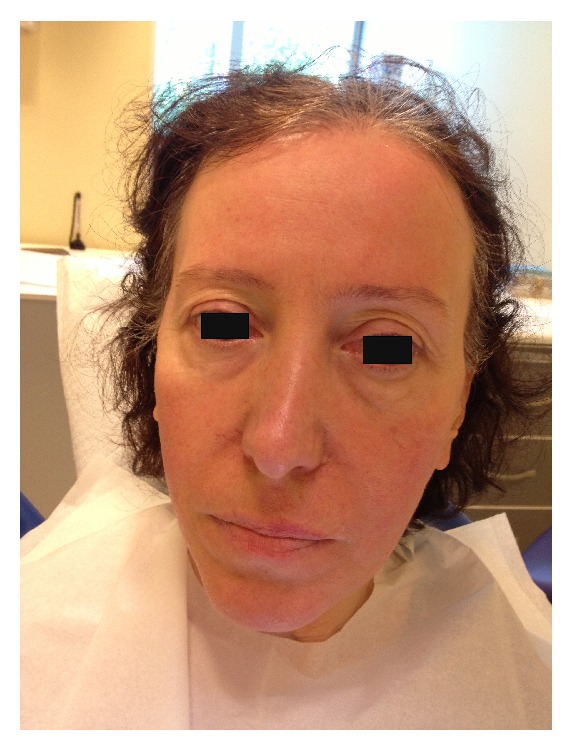
10 days following the incident the patient became asymptomatic.

**Figure 5:**
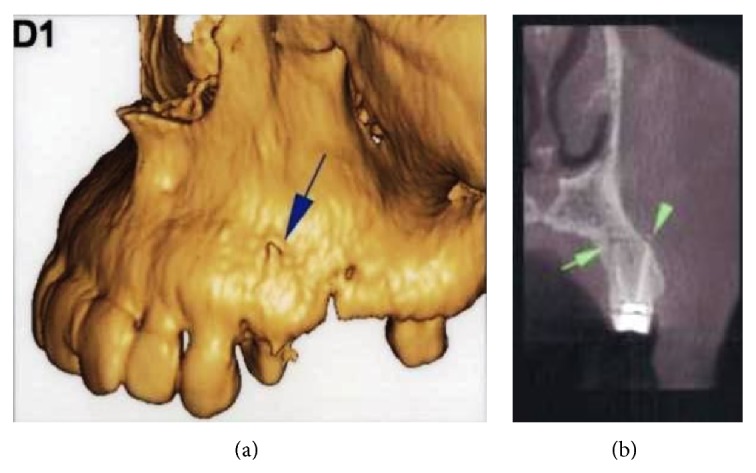
(a) 3D image and (b) coronal view of DVT revealed that the apex of the buccal root canal had perforated the maxillary cortical bone.

**Figure 6:**
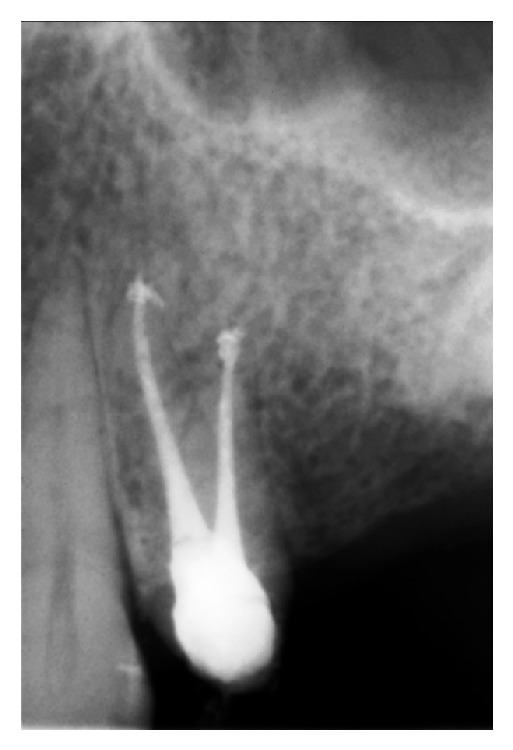
Approximately 4 weeks after the procedure root canals were filled.

The root canals were irrigated with saline solution, and the tooth was restored temporarily. To prevent the risk of infection, intramuscular clindamycin (600 mg twice a day) was administered for three days, and ibuprofen (400 mg) was prescribed for pain management, to be taken as required. Use of cold packs externally for the first day was replaced by warm compresses on the second day for treatment of the swelling. On the first recall, an increase in the ecchymosis was noticed; however, the swelling had decreased significantly (Figure 3). Both swelling and ecchymosis kept progressively decreasing during the follow-up period, and the patient became asymptomatic on the 10th day after the procedure (Figure 4). For the evaluation of the relationship between the tooth, alveolar bone, and the maxillary sinus, dental volumetric tomography (DVT) (Newtom 3G, QR s.r.l., Verona, Italy) was performed. DVT images revealed that the apex of the buccal root canal had perforated the maxillary cortical bone, creating a pathway for the solution into the soft tissues (Figures 5(a) and 5(b)). After 10 days, the symptoms had completely resolved. At this time, a root canal dressing with Ca(OH)_2_ was placed in the canal. At the final appointment approximately 4 weeks after the procedure, the root canals were filled with AH Plus (Dentsply Maillefer) and gutta-percha (Figure 6).

## 3. Discussion

Effective irrigation during chemomechanical shaping is critical to the success of root canal treatment. Irrigation with NaOCl removes biofilm [22] and eliminates the majority of pathogenic microorganisms [17] through its oxidative, hydrolytic, and osmotic effects [14]. However, high concentrations of NaOCl may cause severe tissue injuries [23]. Accurate measurement of the working length is of crucial importance to prevent such unintended consequences. Diagnostic as well as working radiographs should be taken. Use of apex locators in addition to radiographs contributes to precise measurements [24]. Using stoppers on irrigation needles, applying less pressure during irrigation, moving the needle back and forth during irrigation, using side-perforated irrigation needles, and administering lower concentrations of NaOCl decrease the risk of occurrence of these complications [23].

The patient usually feels sudden and severe pain when there is apical extrusion of the irrigating solution, after which swelling, hematoma, and ecchymosis can occasionally be observed [25]. Extrusion into the maxillary sinus should be suspected when the patient reports tastes of chlorine and complains of throat irritation [26, 27]. Severe pain is replaced by constant discomfort as tissue destruction progresses [25]. An apical abscess can develop due to extrusion of infected material in addition to NaOCl into the apical tissues. Mucosal necrosis and long-term paresthesia are possible complications [26, 28]. The relation between the volume or concentration of the NaOCl solution extruding into the soft tissues and the severity of the tissue reaction has not been proven [29]. On the other hand, swelling and ecchymosis may be due to the close relation of vascular structures to the area of apical extrusion [19]. Even though the initial hemorrhage ceases, the area of ecchymosis continues to enlarge due to continuing interstitial leakage. In the current case, the increased ecchymosis observed at the first follow-up appointment can be attributed to this phenomenon.

If there is NaOCl extrusion into the tissues, local anesthesia should be administered for pain relief, and the canals should be irrigated with copious amount of physiologic saline. Measures should be taken to relax the patient and assure him or her that this complication can be controlled. Priority must be given to pain relief, reduction of the swelling, and prevention of secondary infection. The patient should be instructed to use cold compresses during the first few days followed by warm compresses for resolution of the soft tissue swelling and elimination of the hematoma. Analgesics should be prescribed for postoperative pain control [25]. Extrusion of bacterial toxins and bacteria together with NaOCl along with subcutaneous necrotic tissue may induce secondary infection. Thus, prescription of appropriate antibiotics is important. Antihistaminics may be prescribed to prevent allergic reactions [30].

Surgical intervention may be considered in some cases depending on the level of injury and the response to treatment. The goal of surgical intervention is to achieve decompression, ease drainage, and improve prognosis. Hematoma and/or infection may not be restricted within the anatomical borders, as NaOCl causes tissue lysis and spreads along a dimension of its own planes [25]. The majority of the patients experience relief after a few days, following edema, ecchymosis, hematoma, paralysis, and rarely secondary infection. Long-term paresthesia, scars, and aesthetic defects may occur in some cases [27]. There are reports of neurologic injury due to extrusion of NaOCl into soft tissues [28, 31]. There were neither residual tissue defects nor neurologic injuries in the presented case.

The majority of NaOCl extrusions into the periapical area are attributed to incorrect determination of the working length, excessive enlargement of the apical foramen, lateral perforations, needle stuck within the root canal, or vertical root fractures as well [15, 20, 31]. Destruction of periapical alveolar bone due to chronic infection as well as use of high pressure during injection facilitates NaOCl extrusion into soft tissues [32]. In a survey of 23 cases, 18 were female, whereas only 5 were male [19]. In the same report, 20 were in maxillary region and only 3 were in the mandibular jaw. Mandibular teeth are centrally located in more dense cortical bone as compared to maxillary teeth. A thin layer of cortical bone superficially covers the buccal roots of maxillary premolar and molar teeth. Therefore, maxillary teeth are thought to be prone to NaOCl extrusion into soft tissues more than mandibular teeth [19, 33]. Behrents et al. [34] also reported a sodium hypochlorite accident derived from maxillary upper second premolar. With cone beam computed tomography (CBCT) images they demonstrated that the buccal root apex had perforated the buccal plate as was the case with this report. In the present case, chronic infection preexisted periapically, and, additionally, DVT images revealed that the buccal root apex had perforated the maxillary bone, which may have facilitated extrusion of the NaOCl solution. In addition, it is very likely that the operator applied excessive pressure on the needle or wedged the needle in the root canal, making the situation worse. Furthermore, in doubtful cases an initial CBCT would have identified the risk factors for accidents NaOCl.

Some patients may be more sensitive to NaOCl. This issue is supposed to be highlighted during medical anamnesis. The patient should be asked about any history of discomfort due to NaOCl used for household bleach or chlorine in swimming pools before beginning root canal treatment. If there is history of sensitivity, usage of NaOCl must be avoided unless proven that there is no sensitivity provided by the patient. Dermatologic tests for NaOCl are available for chairside application. Irrigation solutions containing either chlorhexidine or ethylenediaminetetraacetic acid (EDTA) should be selected if there are no test results available and there is a previous history of hypochlorous ion sensitivity [34].

Zhu et al. [35] indicated in their review that appearance of facial ecchymosis after NaOCl accident follows the course of superficial venous vasculature. Thus similarity of the locations at which ecchymosis was manifested in the case reports involved in the literature was not an unexpected result. The extent of the ecchymosis is determined by some factors like the amount and concentration of NaOCl entering the venous complex and the specific location of the venous elements and associated tissues [35].

When the literature was reviewed it could be clearly seen that there were limited number of case reports about NaOCl accidents; in fact millions of root canal therapies are performed in a year using NaOCl for irrigation as Behrents et al. [34] have indicated. The authors related this occasion to several conditions that have to exist for developing ecchymosis after NaOCl accident. The apical foramen of the related tooth has to be open to the periapical tissues, an anatomical variation has to exist for drainage of the infused NaOCl directly into the anterior facial vein to result in subcutaneous facial haemorrhage, and apical pressure generated by positive-pressure irrigation delivery system at the periapex has to exceed the venous pressure in the superficial veins of the neck [35]. These factors should be considered during root canal therapy procedures in order to prevent severe NaOCl accidents.

NaOCl accidents may cause a variety of problems, from discoloration of clothes to serious complications requiring surgical intervention. Meticulous attention to detail and efforts to follow irrigation rules are required to prevent such an unexpected and unpleasant event. Syringes as well as NaOCl containers should be labeled to prevent inadvertent injection of NaOCl into the soft tissues instead of anesthetic solution. In a better way anesthetic cartridges should never be filled with any of the irrigation solutions. For cases of NaOCl extrusion into the periradicular area for any reason, measures should be taken to relax the patient, and appropriate treatment following pain management should be initiated. It is reasonable to employ advanced imaging techniques, as they allow 3D visualization of the affected area and may reveal the cause of the incident.
